# Actual and estimated adenoma detection rates: a 2‐year monocentric colonoscopic screening outcome in Shenzhen, China

**DOI:** 10.1002/jgh3.12322

**Published:** 2020-03-16

**Authors:** Li Zeng, Eng G. Chua, Ying Xiong, Shihua Ding, Hui Ai, Zhibo Hou, Mun F. Loke, Khean L. Goh, Chin Y. Tay, Barry J. Marshall, Fuqiang Zhu, Dayong Sun

**Affiliations:** ^1^ Department of Gastroenterology The First Affiliated Hospital of Shenzhen University, The Second People's Hospital of Shenzhen Shenzhen China; ^2^ The Marshall Centre for Infectious Disease Research and Training University of Western Australia Perth Australia; ^3^ Department of Gastroenterology Shenzhen Longhua District Central Hospital Shenzhen China; ^4^ Department of Laboratory Medicine The First Affiliated Hospital of Shenzhen University, The Second People's Hospital of Shenzhen Shenzhen China; ^5^ Kuichong People's Hospital Shenzhen China; ^6^ School of Life Sciences and Chemical Technology Ngee Ann Polytechnic Singapore Singapore; ^7^ Department of Medicine University of Malaya Kuala Lumpur Malaysia; ^8^ Department of Pathology The First Affiliated Hospital of Southern University of Science and Technology, Shenzhen People's Hospital Shenzhen China

**Keywords:** adenoma detection rate, colon, colonoscopy, polyp detection rate

## Abstract

**Background and Aim:**

While adenoma detection rate (ADR) is an important quality metric for screening colonoscopy, it remains difficult to be accessed due to the lack of integrated endoscopy and pathology databases. Hence, the use of an adenoma‐to‐polyp detection rate quotient and polyp detection rate (PDR) has been proposed to predict ADR. This study aimed to examine the usefulness of estimated ADR across different colonic segments in two age groups for Shenzhen people in China.

**Methods:**

We retrospectively analyzed 7329 colonoscopy procedures performed by 12 endoscopists between January 2012 and February 2014. The PDR, actual ADR, and estimated ADR of the entire, proximal, and distal colon, and within each colonic segment, in two patient age groups: <50 and ≥50 years, were calculated for each endoscopist.

**Results:**

The overall polyp and adenoma prevalence rates were 19.1 and 9.3%, respectively. The average age of adenoma‐positive patients was significantly higher than that of adenoma‐negative patients (54 ± 12.6 years *vs* 42.9 ± 13.2 years, respectively). A total of 1739 polyps were removed, among which 826 were adenomas. More adenomatous polyps were found in the proximal colon (60.4%, 341/565) than in the distal colon (40.9%, 472/1154). Overall, both actual and estimated ADR correlated strongly at the entire colon level and within most colonic segments, except for the cecum and rectum. In both age groups, these parameters correlated strongly within the traverse colon and descending colon.

**Conclusion:**

Caution should be exercised when predicting ADR within the sigmoid colon and rectum.

## Graphical abstract

This aim of this retrospective study was to examine the correlations between the actual and estimated adenoma detection rate (ADR) across different colonic segments in two age groups for Shenzhen people in China. The overall polyp and adenoma prevalence were 19.1% and 9.3%, respectively. While the actual and estimated ADR values correlated strongly within the traverse colon and descending colon in both age groups, caution should be exercised when predicting ADR within the sigmoid colon and rectum.

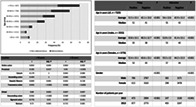

## Introduction

Colorectal cancer (CRC) is the fifth most commonly occurring cancer among the population of China, accounting for nearly 8.8% of total expected cancer cases in 2015 and a mortality rate of about 50.8%.^1^ Colonoscopy screening plays a vital role in the reduction of CRC incidence and mortality rates through the early detection and removal of adenomatous polyps.^2^ While colonoscopy has been considered the gold standard for CRC screening, multiple factors, including bowel preparation, time of withdrawal, and the colonoscopic competency of one endoscopist, could affect patient outcomes.^3^

Adenoma detection rate (ADR), defined as the proportion of individuals who have at least one histologically confirmed adenoma, is an important quality indicator for screening colonoscopy.^3^ Notably, increased ADR has been linked to reduced risk of CRC incidence and deaths, further reflecting the importance of ADR in colonoscopy quality.^4^ Nevertheless, ADR is not readily accessible in many clinical settings as endoscopy findings and pathology results are usually stored separately.

Meanwhile, polyp detection rate (PDR), defined as the proportion of individuals who have at least one polyp detected during a complete screening colonoscopy, can be readily calculated based on endoscopy reports. Importantly, a positive relationship has been reported between PDR and ADR.^5^, ^6^ Hence, the use of a conversion factor, known as the adenoma‐to‐polyp detection rate quotient (APDRQ), to estimate ADR from the endoscopist's PDR has been proposed.^7^ Using this approach, Elhanafi *et al*. demonstrated that, in a predominantly Hispanic population, there is a high correlation between actual ADR and estimated ADR.^8^ Yet, when the correlation was assessed based on individual colonic segments, while strong positive associations were seen in segments proximal to the splenic flexure, significantly weaker correlations were observed in the distal segments, including sigmoid and rectum.^9^

As the PDR and ADR in certain colonic segments do not correlate well with each other, and the adenoma prevalence differs with age, our study aimed to examine the correlation between the actual and estimated ADRs within each colonic segment while providing additional information on PDR and ADR among people in Shenzhen, China.

## Methods

### *Study design*

We retrospectively evaluated screening colonoscopies performed by 12 endoscopists from January 2012 to February 2014 at the Second People's Hospital of Shenzhen. Upon receipt of ethics approval from the research ethics committee (reference number KS20191119006), the endoscopy and pathology results of 11 912 patients were extracted from hospital databases and succinctly reviewed. Patients who had an incomplete colonoscopy (cecal insertion) or poor bowel preparation, previous colorectal surgical operation, no histology examination on detected colonic polyps, or repeated colonoscopies during the study period were excluded from further analysis.

### *Data analysis*

In this study, the proximal colon included the cecum, ascending colon, hepatic flexure, and transverse colon, while the distal colon included the splenic flexure, descending colon, sigmoid, and rectum. Sessile serrated polyps were considered adenomatous polyps in this study given their high risk of malignant transformation. For each endoscopist, PDR and ADR for the entire colon, the proximal and distal colon, and each colonic segment were calculated.

The PDR was calculated by dividing the total number of patients who had at least one polyp detected during a colonoscopy by the total number of patients who underwent a screening colonoscopy. The ADR was calculated by dividing the total number of patients who had at least one histologically confirmed adenoma detected by the total number of patients who underwent a screening colonoscopy. To acquire the averaged group APDRQ conversion factor, the ADR to PDR ratio (for the entire colon) of each endoscopist was summed up and then divided by 12, the total number of endoscopists involved in this study. The estimated ADR was obtained by multiplying the APDRQ conversion factor by PDR.

### *Statistical analysis*

We used the Chi‐square test and unpaired two‐sample Student's *t*‐test for the comparison of categorical and continuous variables, respectively. *P*‐values of less than 0.05 were considered statistically significant following Bonferroni's correction, as appropriate. Pearson's correlation coefficient was used to evaluate the strength of the relationship between estimated and actual ADRs at the endoscopist's level.

## Results

### *Patient characteristics*

**Figure 1:**
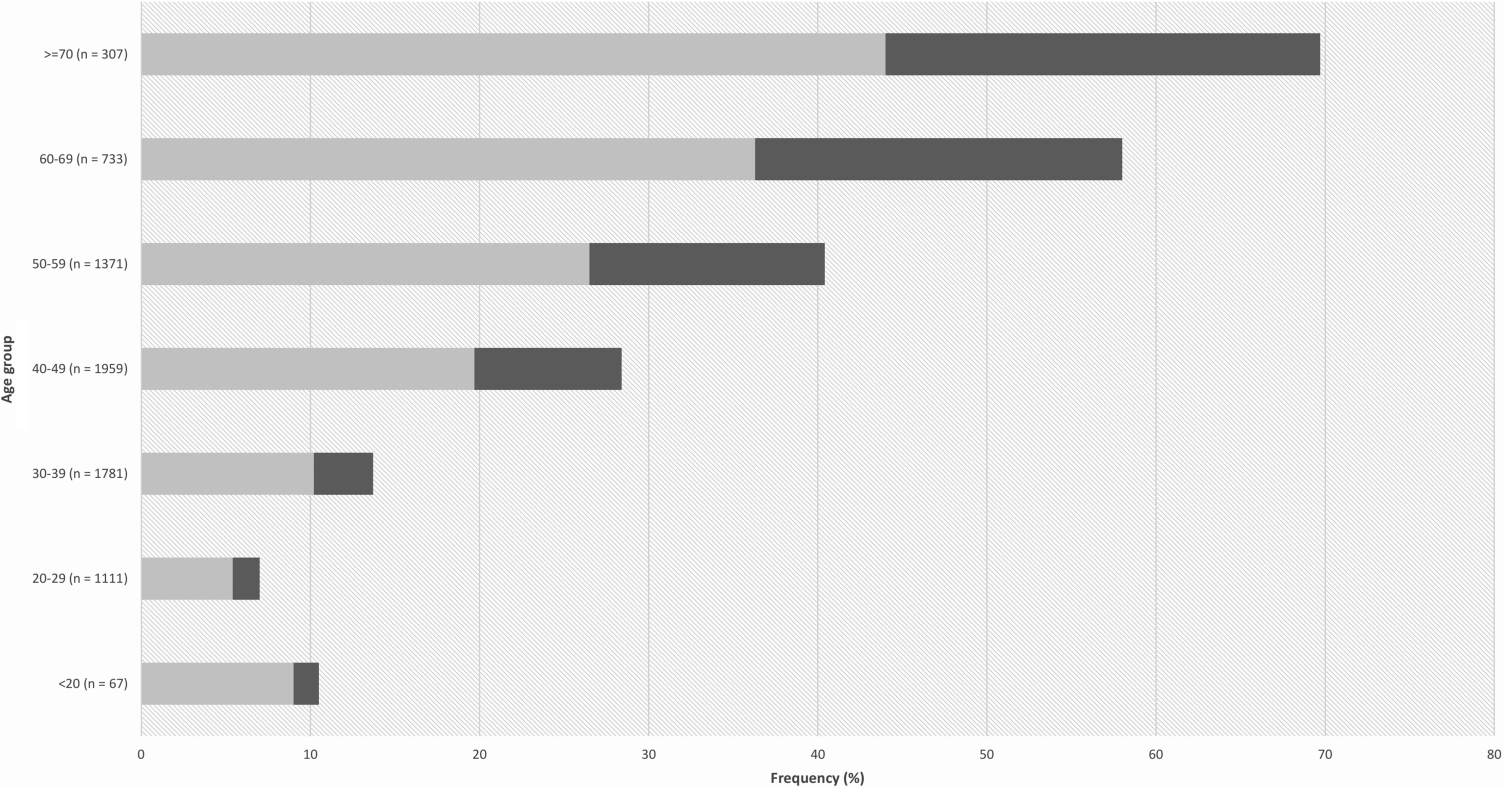
Distribution frequency of nonadenomatous and adenomatous polyps in different age categories. 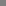, nonadenomatous; 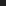, adenomatous.

From January 2012 to February 2014, a total of 11 912 screening colonoscopies were performed by 12 staff endoscopists at the Second People's Hospital of Shenzhen. After application of the exclusion criteria, 7329 colonoscopies were included in the final analysis. Overall, the occurrence of colorectal polyps and adenomas increased with age (Fig. 1). In this study, colorectal polyps were found in 19.1% (1399/7329) of all study participants, among which 680 had adenomas with an overall ADR of 9.3%. The average age of the polyp‐positive cohort was significantly higher compared with that of polyp‐negative patients (51.5 ± 13.1 years *vs* 42.1 ± 13 years, *P* < 0.001) (Table 1). Similarly, a statistically significant age difference was also observed between the adenoma‐positive and ‐negative cohorts (54 ± 12.6 years *vs* 42.9 ± 13.2 years, *P* < 0.001).

**Table 1:** General characteristics of study subjects

	Polyp			Adenoma		
	Positive	Negative	*P*	Positive	Negative	*P*
Age in years (all, *n =* 7329)						
Average	51.5 ± 13.1	42.1 ± 13.0	<0.001	54 ± 12.6	42.9 ± 13.2	<0.001
Median	51	41		55	42	
Age in years (males, *n* = 3553)						
Average	50.8 ± 13.1	40.3 ± 12.7	<0.001	52.8 ± 13	41.4 ± 13.1	<0.001
Median	50	38		53	40	
Age in years (females, *n* = 3776)						
Average	52.4 ± 13.1	43.6 ± 13.1	<0.001	55.5 ± 12	44.2 ± 13.3	<0.001
Median	53	43		57	44	
Gender			<0.001			<0.001
Male	786	2767		382	3171	
Female	613	3163		298	3478	
Number of patients per year						
2012	473	2904	<0.001	247	3130	<0.001
2013	877	2773		403	3247	

Compared to females, males had significantly more polyps and adenomas detected, as well as the tendency to develop a polyp (50.8 ± 13.1 years *vs* 52.4 ± 13.1 years, *P* = 0.025) or adenoma (52.8 ± 13 years *vs* 55.5 ± 13.3 years, *P* = 0.006) at a significantly earlier age. In addition, in 2013, both PDR and ADR (24 and 11%, respectively) were significantly higher compared with that of 2012 (14 and 7.3%, respectively).

### *PDR, actual ADR, and estimated ADR across all patients*

A total of 1739 polyps was found in this study, of which 826 were histologically confirmed adenomas. As depicted in Table 2, more than half of the polyps were detected in the rectum (30.5%, 530/1739) and sigmoid colon (24.5%, 430/1739). Despite the rectum having the highest number of polyps detected in the present study, its proportion of adenomas (31.1%, 165/530) was the lowest compared with that of other colonic segments. It is also important to note that a higher proportion of polyps were adenomatous in the proximal colon (60.4%, 341/565) compared to the distal colon (40.9%, 472/1154).

**Table 2:** Numbers of polyps and adenomas detected in individual colorectal segments

Location	# polyps [(%), *n* = 1739]	# adenomas [(%), *n* = 826]	Adenoma proportion (%)
Proximal	565 (32.5)	341 (41.3)	60.4
Cecum	79 (4.5)	44 (5.3)	55.7
Ascending colon	179 (10.3)	114 (13.8)	63.7
Hepatic flexure	53 (3)	27 (3.3)	50.9
Transverse colon	254 (14.6)	156 (18.9)	61.4
Distal	1154 (66.4)	472 (57.1)	40.9
Splenic flexure	11 (0.6)	6 (0.7)	54.5
Descending colon	183 (10.5)	107 (13)	58.8
Sigmoid colon	430 (24.7)	194 (23.5)	45.1
Rectum	530 (30.5)	165 (20)	31.1
Unspecified	20 (1.2)	13 (1.6)	65

Both PDR and ADR were calculated for each staff endoscopist, as detailed in Table 3. The average PDR was 20.6 ± 7.2% (range: 5.4–33.5%), whereas the average ADR was 10.6 ± 4.2% (range: 2.9–18.5%). In this study, the group APDRQ was 0.512, which was then multiplied by each endoscopist's PDR to obtain individual estimated ADR. The estimated ADR values ranged from 2.8 to 17.2%, and there was a very strong positive correlation between the actual and estimated ADRs throughout the colon (*r* = 0.912, *P* < 0.001).

**Table 3:** Overall PDR and ADR of each staff endoscopist involved in this study

Staff	# procedures	# patients with polyps	# patients with adenomas	PDR	Actual ADR	Estimated ADR
A	446	24	13	5.4	2.9	2.8
B	66	16	9	24.2	13.6	12.4
C	810	196	102	24.2	12.6	12.4
D	1425	246	120	17.3	8.4	8.9
E	367	65	38	17.7	10.4	9.1
F	631	172	96	27.3	15.2	14
G	365	66	42	18.1	11.5	9.3
H	687	128	67	18.6	9.8	9.5
I	1527	216	77	14.1	5	7.2
J	171	35	16	20.5	9.4	10.5
K	233	78	43	33.5	18.5	17.2
L	601	157	57	26.1	9.5	13.4
Total	7329	1399	680			

ADR, adenoma detection rate; PDR, polyp detection rate.

Next, we assessed the relationship between the actual and estimated ADRs of each individual colonic segment, except for the splenic flexure as there were multiple zero values in the data and might therefore produce a spurious correlation. In general, a greater association level was demonstrated between the values derived from the proximal colon (*r* = 0.921) and those from the distal colon (*r* = 0.872) (Table 4). The colonic segments that displayed the highest correlations were the ascending colon, traverse colon, and descending colon, with *r* values of 0.932, 0.947, and 0.94, respectively. Within the cecum and rectum, however, only moderate correlation levels were observed.

**Table 4:** Pearson's correlation between the actual and estimated ADRs of different colonic segments across all patients

Location	*r*	Adj. *P*
Entire colon	0.912	<0.001
Proximal	0.921	<0.001
Cecum	0.647	0.23
Ascending colon	0.932	<0.001
Hepatic flexure	0.838	0.007
Transverse colon	0.947	<0.001
Distal	0.872	0.002
Descending colon	0.94	<0.001
Sigmoid colon	0.813	0.013
Rectum	0.722	0.08

ADR, adenoma detection rate.

### *Comparison of actual and estimated ADRs in two patient age groups: <50 and ≥50 years*

As the average age of our ADR‐positive patients was greater than 50 years, a minimum age cut‐off of 50 years was selected to divide our patients into two groups, one consisting of 4918 patients younger than 50 years of age and the other consisting of 2411 patients aged 50 years and older. We then assessed and compared between these two groups the relationships between actual ADRs and estimated ADRs per each colonic segment based on newly acquired ADPRQ values of 0.396 and 0.581, respectively. All PDR, actual ADR, and estimated ADR values for this section of analysis are given in Table S1. As shown in Table 5, significantly higher proportions of polyps and adenomas were detected in nearly every colonic segment in patients aged 50 years and older. Pearson correlation analysis demonstrated significantly weaker association outcomes in the proximal colon of patients younger than than 50 years old compared to the older patients (*r* values of 0.585 and 0.94, respectively) (Table 6). Notably, poor to fair association outcomes were seen among younger patients within the cecum (*r* = 0.179) and ascending colon (*r* = 0.404), respectively. In both younger and older patients, while both actual and estimated ADRs correlated very strongly within the traverse colon (*r* values of 0.942 and 0.936, respectively) and descending colon (*r* values of 0.943 and 0.949, respectively), moderate association levels were observed within the sigmoid colon (*r* values of 0.776 and 0.707, respectively) and rectum (*r* values of 0.72 and 0.721, respectively).

**Table 5:** Comparison of PDR and ADR within different colonic segments among two age categories

	# patients with polyps			# patients with adenomas		
	<50 years	≥50 years		<50 years	≥50 years	
Location	(*n* = 4918)	(*n* = 2411)	Adj. *P*	(*n* = 4918)	(*n* = 2411)	Adj. *P*
Entire colon	634 (12.9)	765 (31.7)	<0.001	251 (5.1)	429 (17.8)	<0.001
Proximal	206 (4.2)	315 (13.1)	<0.001	107 (2.2)	208 (8.6)	<0.001
Cecum	37 (0.8)	40 (1.7)	0.004	21 (0.4)	23 (1)	0.067
Ascending colon	67 (1.4)	110 (4.6)	<0.001	34 (0.7)	80 (3.3)	<0.001
Hepatic flexure	21 (0.4)	32 (1.3)	<0.001	8 (0.2)	19 (0.8)	<0.001
Transverse colon	92 (1.9)	158 (6.6)	<0.001	50 (1)	105 (4.4)	<0.001
Distal	471 (9.6)	550 (22.8)	<0.001	159 (3.2)	268 (11.1)	<0.001
Splenic flexure	5 (0.1)	6 (0.2)	1	4 (0.1)	2 (0.1)	1
Descending colon	74 (1.5)	108 (4.5)	<0.001	38 (0.8)	69 (2.9)	<0.001
Sigmoid colon	187 (3.8)	241 (10)	<0.001	73 (1.5)	121 (5)	<0.001
Rectum	257 (5.2)	266 (11)	<0.001	57 (1.2)	108 (4.5)	<0.001

ADR, adenoma detection rate; PDR, polyp detection rate.

**Table 6:** Pearson's correlation between the actual and estimated ADRs of different colonic segments among two patient age groups

	<50 years		≥50 years	
Location	*r*	Adj. *P*	*r*	Adj. *P*
Entire colon	0.853	0.004	0.863	0.003
Proximal	0.585	0.457	0.94	<0.001
Cecum	0.179	1	0.841	0.006
Ascending colon	0.404	1	0.905	<0.001
Hepatic flexure	0.849	0.005	0.691	0.128
Transverse colon	0.942	<0.001	0.936	<0.001
Distal	0.88	0.002	0.837	0.007
Descending colon	0.943	<0.001	0.949	<0.001
Sigmoid colon	0.776	0.03	0.707	0.101
Rectum	0.72	0.083	0.721	0.081

## Discussion

In this study, the overall polyp and adenoma prevalence rates were 19.1 and 9.3%, respectively, which were comparable to the findings of another study recently conducted in Wenzhou City in Southeast China, at 23.9 and 13.3%, respectively.^10^ Interestingly, when comparing the overall adenoma prevalence in this study to that of our neighboring countries, while Taiwan had a slightly increased rate of 16.1%, Korean individuals were at least thrice as likely to be detected with adenoma (29.1%).^11^, ^12^ The substantially higher incidence of colorectal adenoma in Koreans, despite both China and Korea being in close proximity to each other within the East Asia region, might be related to genetic differences or variations in lifestyle and dietary intake.

The average ages of both polyp‐positive and adenoma‐positive subjects were 51.5 ± 13.1 years and 54 ± 12.6 years, respectively, suggesting that the appropriate starting age for CRC screening should be 50 years old. This finding is in agreement with the latest diagnosis and treatment guidelines for CRC released in 2018 by The Chinese Society of Clinical Oncology and with several international CRC screening guidelines, in which CRC screening in average‐risk subjects should be performed at the age of 50 years and older.^13^, ^14^ Consistent with several previous reports, our study showed that adenoma prevalence increased with age, and men were at significantly greater risk of developing adenoma than women.^10^, ^15^, ^16^

Our findings indicated that, although more polyps were more commonly found in the distal colon compared to the proximal colon, the likelihood of one or more polyp(s) being adenomatous was significantly higher in the latter than the former. This observation is in line with Hong *et al*. who also previously showed that a higher proportion of polyps in the Chinese population was adenomatous in the proximal colon relative to the distal colon.^10^ It has been reported that proximal tumors were often more microsatellite unstable and hypermutated compared to the distal tumors, suggesting that the DNA repair mechanisms within the proximal colon epithelial cells might be intrinsically less efficient compared to the distal colon counterparts and thus explaining why proximal colon is more prone to adenomas that have increased risk of developing into CRC.^17^

The use of screening colonoscopy for early detection and removal of adenomas has been proven important in the reduction of CRC incidence and mortality.^2^, ^18^, ^19^, ^20^ Of several quality measures established to ensure high‐quality colonoscopy, ADR is the most extensively used metric.^21^ It has been shown that an increase of 1% in the ADR could lead to 3% CRC risk reduction.^22^ Based on the recommendation by The American College of Gastroenterology, the overall ADR in averaged‐risk individuals aged 50 years and older should be at least 25%.^23^ However, our study found only 17.8% in the older patient group, below the recommended level. This finding emphasizes the critical need of providing further training to endoscopists who had individual ADR of less than 25% to improve colonoscopy performance. In addition to inadequate operator skills, suboptimal ADR could also be related to short withdrawal time. Thus, prolonging the observation time to 6 min or more to improve ADR, as previously reported, might be worth considering.^24^

As it is difficult to obtain actual ADR due to the lack of integrated databases containing both endoscopy and histology reports, the use of a group‐averaged ADPRQ conversion factor to estimate ADR from PDR has been proposed.^7^ Based on this approach, we found that there is a very strong positive association between actual and estimated ADR values for the entire colon. However, when these parameters were analyzed per segment, insignificant correlations were observed in the cecum and rectum. Further correlation analysis across two separate patient age groups demonstrated comparable outcomes within the rectum in both groups, indicating that the difference between actual and estimated ADRs within this segment is not age‐dependent but is rather due to the intrinsic lower incidence of adenomas in this region compared to other colonic segments. Compared to the younger patients, both actual and estimated ADRs were correlated to a lesser extent within the sigmoid colon of the older patient group. This might be due to the increased likelihood of significant fixation and narrowing of the sigmoid colon as a result of diverticular disease and thus the increased difficulty of polyp detection in the older patients.^25^

On the other hand, while the actual and the estimated ADRs were strongly correlated within the cecum and ascending colon of patients aged 50 years and older, poor to fair associations were indicated in the younger counterparts, respectively. Such findings, however, were in fact because of two endoscopists whose segmental PDR and ADR values were only fairly correlated with each other in the younger patients, as part of their low procedure volume in this study. Importantly, the presence of these unreliable values, in a few, could have a substantial dampening effect on the relationship between both parameters within the cecum and ascending colon of patients younger than 50 years old. Furthermore, in the absence of these parameters from those two endoscopists, the correlation coefficient value improved drastically from 0.179 to 0.819 within the cecum and from 0.404 to 0.937 within the ascending colon. Together, caution is recommended when dealing with estimated ADR as it requires a high degree of correlation between the PDR and ADR of participating endoscopists.

In summary, the average age of adenoma‐positive subjects in this study fulfills the CRC screening age guideline set in mainland China and several Western countries. Older age and male gender are both risk factors for colonic adenomas. More polyps were adenomatous in the proximal colon relative to the distal colon. The ADR can be confidently estimated from PDR within the traverse colon and descending colon in all patients, regardless of age. Caution should be exercised when predicting ADR for the rectum and sigmoid colon, and it is important to use highly correlated PDR and ADR data for more accurate ADR prediction prior to its use as a colonoscopy quality metric.

## Supporting information


**Table S1.** PDR, actual ADR, and estimated ADR values per colonic segment across two patient age groups.
